# Supplementary material for: A reconfigurable arbitrary retarder array as complex structured matter
Source: Nat Commun. 2025 May 27;16:4902. doi: 10.1038/s41467-025-59846-4 (PMC12117100; doi:10.1038/s41467-025-59846-4)
Supplement: Supplementary file 1 — Supplementary Information [file 41467_2025_59846_MOESM1_ESM.pdf]

Supplementary Information

A reconfigurable arbitrary retarder array as complex structured matter

Chao He[1,†,*], Binguo Chen[2,†], Zipei Song[1,†], Zimo Zhao[1,†], Yifei Ma[1,†], Honghui He[2,*], Lin Luo[3,*], Tade Marozsak[1], An Aloysius Wang[1], Rui Xu[3], Peixiang Huang[3], Jiawen Li[2], Xuke Qiu[1], Yunqi Zhang[1], Bangshan Sun[1], Jiahe Cui[1], Yuxi Cai[1], Yun Zhang[4], Andong Wang[1], Mohan Wang[1], Patrick Salter[1], Julian AJ Fells[1], Ben Dai[5], Shaoxiong Liu[6], Limei Guo[7], Yonghong He[2], Hui Ma[2], Daniel J Royston[8,9], Steve J Elston[1], Qiwen Zhan[10], Chengwei Qiu[11], Stephen M Morris[1], Martin J Booth[1], and Andrew Forbes[12]

[1]Department of Engineering Science, University of Oxford, Parks Road, Oxford, OX1 3PJ, UK
[2]Institute of Biopharmaceutical and Health Engineering, Tsinghua Shenzhen International Graduate School, Tsinghua University, Shenzhen 518055, China
[3]College of Engineering, Peking University, Beijing 100871, China
[4]Key Laboratory of Archaeological Sciences and Cultural Heritage, Chinese Academy of Social Sciences, Beijing, 102488, China
[5]Department of Statistics, The Chinese University of Hong Kong, Shatin, HK SAR, China
[6]Shenzhen Sixth People's Hospital, Huazhong University of Science and Technology Union Shenzhen Hospital, 518052 Shenzhen, China
[7]Department of Pathology, School of Basic Medical Science, Peking University Health Science Center, Peking University Third Hospital, Beijing, China
[8]Nuffield Division of Clinical Laboratory Sciences, Radcliffe Department of Medicine, University of Oxford, Oxford, UK
[9]Department of Pathology, Oxford University Hospitals NHS Foundation Trust, Oxford, UK
[10]School of Optical-Electrical and Computer Engineering, University of Shanghai for Science and Technology, Shanghai 200093, China
[11]Department of Electrical and Computer Engineering, National University of Singapore, Singapore 117583, Singapore
[12]School of Physics, University of the Witwatersrand, Private Bag 3, Johannesburg 2050, South Africa

[†]These authors contributed equally to this work
[*]Corresponding authors: chao.he@eng.ox.ac.uk; he.honghui@sz.tsinghua.edu.cn; luol@pku.edu.cn

Supplementary Method 1: Tuneable arbitrary elliptical retarder by cascaded optical elements

This section discusses how pixel-controlled, low functionality tuneable devices can be cascaded to behave equivalently to an arbitrary elliptical retarder array, and fully control the state of polarisation (SoP) and the phase of a light beam (arbitrary to arbitrary conversion). One such setup consists of three spatial light modulators (SLMs) followed by a deformable mirror. Let the SLMs (denoted by A, B, and C) have fast axis orientations ($\vec{S}_n$) of horizontal, diagonal and horizontal, respectively:

$$\vec{S}_A = \begin{bmatrix}1\\0\\0\end{bmatrix} \qquad \vec{S}_B = \begin{bmatrix}0\\1\\0\end{bmatrix} \qquad \vec{S}_C = \begin{bmatrix}1\\0\\0\end{bmatrix} \tag{1}$$

This SLM cascade can mimic a phase-insensitive elliptical retarder at the pixel level, with any desired fast (and slow) axis orientation and retardance value. By introducing a deformable mirror into the system, full control of the absolute phase of the light field can be achieved in the spatial domain.

As mentioned before, the SoP is only affected by the retarders. Each of them rotates the Poincaré sphere around its fast axis by an angle equal to its retardance value. It is then sufficient to decompose an arbitrary rotation into a sequence of three about a fixed axis orientation. To understand why this is always possible, let us consider an arbitrary rotation $\mathbf{R} \in \mathrm{SO}(3)$, represented by an orthogonal 3×3 matrix with $\det \mathbf{R} = 1$. Let us denote the unit vectors in the x, y, z directions by $\hat{\mathbf{x}}, \hat{\mathbf{y}}, \hat{\mathbf{z}} \in \mathbb{R}^3$, and rotations about them by $\mathbf{R}_{\hat{\mathbf{x}}}, \mathbf{R}_{\hat{\mathbf{y}}}, \mathbf{R}_{\hat{\mathbf{z}}} \in \mathrm{SO}(3)$.

Suppose that $\hat{\mathbf{x}}$ is rotated to the point $\hat{\mathbf{p}} = \mathbf{R}\hat{\mathbf{x}}$. It is always possible to transform from one point on the unit-sphere to another one by carrying out a sequence of two rotations with orthogonal axes. Hence, for any $\mathbf{R}$ there exist θ_1, θ_2 in the interval $[-\pi, +\pi]$ such that

$$\mathbf{R}_{\hat{\mathbf{y}}}(\theta_2)\mathbf{R}_{\hat{\mathbf{x}}}(\theta_1)\hat{\mathbf{p}} = \hat{\mathbf{x}} \quad \Leftrightarrow \quad [\mathbf{R}_{\hat{\mathbf{y}}}(\theta_2)\mathbf{R}_{\hat{\mathbf{x}}}(\theta_1)\mathbf{R}]\,\hat{\mathbf{x}} = \mathbf{R}'\hat{\mathbf{x}} = \hat{\mathbf{x}}$$

Where $\mathbf{R}' \in \mathrm{SO}(3)$ represents a rotation. However, any $\mathbf{R}'$ satisfying $\mathbf{R}'\hat{\mathbf{x}} = \hat{\mathbf{x}}$ must describe a rotation around $\hat{\mathbf{x}}$ by an angle θ_3 in the range $-\pi < \theta_3 \leq \pi$. Therefore it holds that

$$\mathbf{R}_{\hat{\mathbf{y}}}(\theta_2)\mathbf{R}_{\hat{\mathbf{x}}}(\theta_1)\mathbf{R} = \mathbf{R}_{\hat{\mathbf{x}}}(\theta_3) \quad \Leftrightarrow \quad \mathbf{R} = \mathbf{R}_{\hat{\mathbf{x}}}^{-1}(\theta_1)\mathbf{R}_{\hat{\mathbf{y}}}^{-1}(\theta_2)\mathbf{R}_{\hat{\mathbf{x}}}(\theta_3)$$

This proves that any $\mathbf{R}$ can be expressed as a sequence of rotations about the $x - y - x$ directions with angles between $[-\pi, +\pi]$, or equivalently over any interval of length 2π.

To explicitly compute such a decomposition, (not necessarily unique) , we can use quaternions to find expressions for the system's overall axis and retardance, as well as the retardance values of the cascaded SLMs. Quaternions are mathematical structures extending complex numbers to four dimensions, and the unit quaternions, as a double cover of SO(3), are often used to represent rotations in three-dimensions. A rotation by angle θ around the axis can be defined by the unit vector (a pure unit quaternion)

$$\mathbf{u} = \begin{pmatrix} 0, & u_x, & u_y, & u_z \end{pmatrix} = u_x i + u_y j + u_z k \tag{2}$$

and can be represented by conjugation through a unit quaternion $\mathbf{q}$

$$\mathbf{q} = \cos\left(\frac{\theta}{2}\right) + \sin\left(\frac{\theta}{2}\right)(u_x i + u_y j + u_z k) \tag{3}$$

Consider a series of rotations about x by θ_1, y by θ_2, and x by θ_3 (such transformations can be achieved by using retarders with corresponding axis orientations and retardance values). Using quaternions, the resulting rotation can be obtained from the product:

$$\mathbf{q}_1\mathbf{q}_2\mathbf{q}_3 = \left[\cos\left(\frac{\theta_1}{2}\right) + \sin\left(\frac{\theta_1}{2}\right) i\right] \cdot \left[\cos\left(\frac{\theta_2}{2}\right) + \sin\left(\frac{\theta_2}{2}\right) j\right] \cdot \left[\cos\left(\frac{\theta_3}{2}\right) + \sin\left(\frac{\theta_3}{2}\right) i\right] \tag{4}$$

Multiplying out and equating with the unit quaternion corresponding to the rotation induced by an elliptical retarder with fast axis $\mathbf{S} = S_1 i + S_2 j + S_3 k$ and retardance θ_S, we get

$$\cos\left(\frac{\theta_2}{2}\right)\cos\left(\frac{\theta_1+\theta_3}{2}\right) = \cos\left(\frac{\theta_S}{2}\right) \tag{5}$$

$$\sin\left(\frac{\theta_1+\theta_3}{2}\right)\cos\left(\frac{\theta_2}{2}\right) i = S_1 \sin\left(\frac{\theta_S}{2}\right) i \tag{6}$$

$$\sin\left(\frac{\theta_2}{2}\right)\cos\left(\frac{\theta_1-\theta_3}{2}\right) j = S_2 \sin\left(\frac{\theta_S}{2}\right) j \tag{7}$$

$$\sin\left(\frac{\theta_2}{2}\right)\sin\left(\frac{\theta_1-\theta_3}{2}\right) k = S_3 \sin\left(\frac{\theta_S}{2}\right) k \tag{8}$$

where $\mathbf{S}$ is the normalised Stokes vector of the retarder's eigen-axis. For $0 \leq \theta_2 < 2\pi$ and $\theta_2 \neq l\pi,\ l \in \mathbb{Z}$ (which corresponds to a horizontally oriented fast axis, retardance value of π). We then have

$$\frac{\theta_1+\theta_3}{2} = \arctan 2\left[\cos\left(\frac{\theta_S}{2}\right), S_1 \sin\left(\frac{\theta_S}{2}\right)\right] + n\pi,\ n \in \mathbb{Z} \tag{9}$$

$$\frac{\theta_1-\theta_3}{2} = 2\arctan 2\left[S_2 \sin\left(\frac{\theta_S}{2}\right), S_3 \sin\left(\frac{\theta_S}{2}\right)\right] + m2\pi,\ m \in \mathbb{Z} \tag{10}$$

From Equation 9 and Equation 10 we can recover two distinct sets of solutions for θ_1 and θ_3 both in the range $[0, 2\pi]$. A closed form solution of θ_2 (angle of rotation about y) is then given by

$$\theta_2 = 2\arctan 2\left[\frac{\cos\left(\frac{\theta_S}{2}\right) + S_1 \sin\left(\frac{\theta_S}{2}\right)}{\cos\left(\frac{\theta_1+\theta_3}{2}\right) + \sin\left(\frac{\theta_1+\theta_3}{2}\right)}, \frac{S_2 \sin\left(\frac{\theta_S}{2}\right) + S_3 \sin\left(\frac{\theta_S}{2}\right)}{\cos\left(\frac{\theta_1-\theta_3}{2}\right) + \sin\left(\frac{\theta_1-\theta_3}{2}\right)}\right] \tag{11}$$

which gives one solution in the interval $[0, 2\pi]$ for each set of $\theta_{1,2}$ (only differing in sign). For $\theta_2 = l\pi,\ l \in \mathbb{Z}$ we can assign

$$\begin{aligned} \theta_1 &= \theta_S \\ \theta_2 &= 0 \\ \theta_3 &= 0 \end{aligned} \tag{12}$$

These expressions deduce values $\theta_{1,2,3}$ for an arbitrary rotation of the Poincaré sphere, corresponding to the action of how three SLMs should behave with respect to any phase-insensitive elliptical retarder.

Building on this foundation, there are different strategies for calculating the phase profiles on the SLMs depending on the input and output beam profiles. For uniform input and non-uniform output beam profiles, two-SLM-based

methods [20] can provide a unique phase pattern solution (with retardance values from 0 to 2π and 0 to π) while keeping the third SLM flat. These unique patterns are then applied to the two SLMs, as demonstrated in most of the experimental results shown in main article Fig. 2a. For non-uniform input and output profiles, all three SLMs must be used, but the phase patterns solutions are non-unique. In this case, criteria such as minimal retardance should be applied to select the optimal phase solution. Notably, the three-SLM strategy can also be applied to uniform input cases, as shown in Fig. 2b.

Supplementary Method 2: Calibration process for retardance modulation in SLM

To precisely relate the retardance value to the applied voltage for optimal SoP control, each pixel of the SLM needs to be calibrated. The calibration begins by positioning the SLM between two orthogonally aligned polarisers, both at a 45° angle to the liquid crystal director. This configuration allows for pixelated intensity modulation via the Fréedericksz transition of the nematic liquid crystal (NLC) within the SLM. The transmittance of light through the NLC layer is described by the equation:

$$T = \sin^2\left(\frac{\pi \Delta n d}{\lambda}\right) \tag{13}$$

where Δn represents the birefringence of the NLC, d is thickness of the device, and λ is the wavelength of the light. The retardance ϕ can be expressed as:

$$\phi = \frac{\pi \Delta n d}{\lambda} \tag{14}$$

Therefore, the retardance ϕ can be derived from the measured intensity as:

$$\phi = \sin^{-1}\left(\sqrt{T}\right) \tag{15}$$

Voltage is applied to each pixel of the SLM to adjust birefringence, with pixel intensities mapped to grayscale values using a manufacturer-defined grayscale-to-voltage map. The calibration process involves incrementally applying flat grayscale values (0-255) across the entire SLM and capturing images with a monochrome camera. These images map the retardance modulation response of each pixel across the grayscale range. By identifying the grayscale range where the SLM achieves a full range of 2π modulation, a one-to-one mapping between pixel values and modulated retardance is generated during the calibration, which are encoded into the look-up table (LUT) for precise phase control. For the modulation higher than 2π, the phase can be wrapped back into the modulation range of the SLM due to the periodic property of the phase.

To evaluate the performance of the calibration procedure, we introduce two important criteria: vectorial precision (P) and vectorial uniformity (U). Vectorial precision is defined as the norm of the difference between the target SoP and the actual SoP on the Poincaré sphere. Vectorial uniformity is the standard deviation of the actual SoP from the mean SoP across the entire field. Further details on these criteria can be found in Ref [17].

Supplementary Method 3: Topological protection of the skyrmionic beams when passing through uniform complex media

In this section, we give mathematical proof of the topological protection properties of skyrmionic beams when passing through complex media. Heuristically, this protection comes from the homotopy invariance of the degree of a continuous function but can also be explicitly computed in certain cases. We show the result in the general anisotropic case. The isotropic case, as a special subset of anisotropic media, then follows immediately. Let us begin with an arbitrary retarder. The Jones matrix of an arbitrary retarder can be characterized by its eigenvectors and eigenvalues, which represent two unique orthogonal axes and the corresponding phase shift between them. In general, the Jones vector of a beam can be represented as:

$$E = \begin{bmatrix} \cos\alpha \\ \sin\alpha e^{i\delta} \end{bmatrix} \tag{16}$$

The Jones matrix of an arbitrary retarder can be represented as:

$$
\begin{aligned}
\mathbf{J}_{\mathrm{AR}} &= \begin{bmatrix} \cos\alpha & -\sin\alpha e^{-i\delta} \\ \sin\alpha e^{i\delta} & \cos\alpha \end{bmatrix} \begin{bmatrix} e^{i\psi+i\phi/2} & 0 \\ 0 & e^{i\psi-i\phi/2} \end{bmatrix} \begin{bmatrix} \cos\alpha & \sin\alpha e^{-i\delta} \\ -\sin\alpha e^{i\delta} & \cos\alpha \end{bmatrix} \\
&= e^{i\psi} \begin{bmatrix} e^{i\phi/2}\cos^2\alpha + e^{-i\phi/2}\sin^2\alpha & (e^{i\phi/2} - e^{-i\phi/2})\sin\alpha\cos\alpha e^{-i\delta} \\ (e^{i\phi/2} - e^{-i\phi/2})\sin\alpha\cos\alpha e^{i\delta} & e^{i\phi/2}\sin^2\alpha + e^{-i\phi/2}\cos^2\alpha \end{bmatrix} \\
&= e^{i\psi}\mathrm{SO3}(\delta, \phi, \alpha)
\end{aligned} \tag{17}
$$

where $e^{i\psi}$ in $\mathbf{J}_{\mathrm{AR}}$ represents the absolute phase introduced by the medium. Since our focus is on SoP rather than phase profile, this uniform phase shift factor will be omitted in the following discussion. Note that from the expression of the skyrmion number, it is clear that the absolute phase does not change the topology of the optical field. In Mueller-Stokes formalism, the incident beam in Equation 16 can be represented as:

$$
S = \begin{bmatrix} s_0 \\ s_1 \\ s_2 \\ s_3 \end{bmatrix} = \begin{bmatrix} 1 \\ \cos 2\alpha \\ \sin 2\alpha \cos\delta \\ \sin 2\alpha \sin\delta \end{bmatrix} \tag{18}
$$

By replacing the eigenvector terms in $\mathbf{J}_{\mathrm{AR}}$ with Stokes vector notations, we can get the Jones matrix of an arbitrary retarder expressed with Stokes parameters:

$$
\mathbf{J}_{\mathrm{AR}} = \begin{bmatrix} \cos(\phi/2) + i*\sin(\phi/2)s_1 & \sin(\phi/2)(i*s_2 + s_3) \\ \sin(\phi/2)(i*s_2 - s_3) & \cos(\phi/2) - i*\sin(\phi/2)s_1 \end{bmatrix} \tag{19}
$$

Furthermore, we can obtain the Mueller matrix of an arbitrary retarder from the Jones matrix by:

$$
\begin{aligned}
\mathbf{M}_{\mathrm{AR}} &= \mathbf{A}(\mathbf{J}_{\mathrm{AR}} \otimes \mathbf{J}^*_{\mathrm{AR}})\mathbf{A}^{-1} \\
&= \begin{bmatrix} 1 & 0 & 0 & 0 \\ 0 & s_1^2(1-\cos\phi) + \cos\phi & s_1 s_2(1-\cos\phi) + s_3\sin\phi & s_1 s_3(1-\cos\phi) - s_2\sin\phi \\ 0 & s_1 s_2(1-\cos\phi) - s_3\sin\phi & s_2^2(1-\cos\phi) + \cos\phi & s_2 s_3(1-\cos\phi) + s_1\sin\phi \\ 0 & s_1 s_3(1-\cos\phi) + s_2\sin\phi & s_2 s_3(1-\cos\phi) - s_1\sin\phi & s_3^2(1-\cos\phi) + \cos\phi \end{bmatrix} \\
&= \begin{bmatrix} 1 & \mathbf{0} \\ \mathbf{0}^T & \mathbf{m}_R \end{bmatrix}
\end{aligned} \tag{20}
$$

where $\otimes$ represents the Kronecker product, $\mathbf{J}^*_{\mathrm{AR}}$ represents the complex conjugate of the above Jones matrix and A is in the form of:

$$
\mathbf{A} = \begin{bmatrix} 1 & 0 & 0 & 1 \\ 1 & 0 & 0 & -1 \\ 0 & 1 & 1 & 0 \\ 0 & i & -i & 0 \end{bmatrix} \tag{21}
$$

Note that the sub-matrix $\mathbf{m}_R$ of M_{AR} is actually a special orthogonal matrix, which can also be derived from Rodrigues' rotation formula. The physical meaning of the sub-matrix $\mathbf{m}_R$ suggests that for any given Stokes vector represented on the Poincaré sphere, the sub-matrix $\mathbf{m}_R$ corresponds to a rotation along the axis given by $S = [s_1, s_2, s_3]^T$ with angle ϕ on the sphere.

Full Poincaré beams have been used to represent a skyrmionic field with a determined skyrmion number. To show the topological protection property of such a beam, we prove below that the skyrmion number does not change after passing through an arbitrary retarder.

The skyrmion number of an optical skyrmion can be expressed as [2, 5]:

$$
S = \frac{1}{4\pi}\iint_\sigma \mathbf{n} \cdot \left(\frac{\partial \mathbf{n}}{\partial x} \times \frac{\partial \mathbf{n}}{\partial y}\right) dxdy \tag{22}
$$

We denote by $\mathbf{n}' = [s'_1, s'_2, s'_3]$ the Stokes vector corresponding to $\mathbf{n} = [s_1, s_2, s_3]$ after passing through the arbitrary retarder $\mathbf{m}_R$. The skyrmion number of this Stokes vector field can be represented as:

$$
\begin{aligned}
S' &= \frac{1}{4\pi}\iint_\sigma \mathbf{n}' \cdot \left(\frac{\partial \mathbf{n}'}{\partial x} \times \frac{\partial \mathbf{n}'}{\partial y}\right) dxdy \\
&= \frac{1}{4\pi}\iint_\sigma \mathbf{m}_R\mathbf{n} \cdot \left(\frac{\partial \mathbf{m}_R\mathbf{n}}{\partial x} \times \frac{\partial \mathbf{m}_R\mathbf{n}}{\partial y}\right) dxdy
\end{aligned} \tag{23}
$$

Considering that $\mathbf{m}_R$ is independent of x and y, we have:

$$\begin{aligned}\frac{\partial \mathbf{m}_R\mathbf{n}}{\partial x} &= \mathbf{m}_R\frac{\partial \mathbf{n}}{\partial x}\\ \frac{\partial \mathbf{m}_R\mathbf{n}}{\partial y} &= \mathbf{m}_R\frac{\partial \mathbf{n}}{\partial y}\end{aligned} \tag{24}$$

Since $\mathbf{m}_R$ is a rotation matrix with $\det(\mathbf{m}_R) = 1$, the skyrmion density can be simplified into:

$$\begin{aligned}\mathbf{m}_R\mathbf{n}\cdot\left(\frac{\partial \mathbf{m}_R\mathbf{n}}{\partial x}\times\frac{\partial \mathbf{m}_R\mathbf{n}}{\partial y}\right) &= \mathbf{m}_R\mathbf{n}\cdot\left(\mathbf{m}_R\frac{\partial \mathbf{n}}{\partial x}\times\mathbf{m}_R\frac{\partial \mathbf{n}}{\partial y}\right)\\ &= (\mathbf{m}_R\mathbf{n})\cdot\mathbf{m}_R\left(\frac{\partial \mathbf{n}}{\partial x}\times\frac{\partial \mathbf{n}}{\partial y}\right)\\ &= \left(\mathbf{n}^T\mathbf{m}_R^T\mathbf{m}_R\right)\left(\frac{\partial \mathbf{n}}{\partial x}\times\frac{\partial \mathbf{n}}{\partial y}\right)\\ &= \mathbf{n}^T\left(\frac{\partial \mathbf{n}}{\partial x}\times\frac{\partial \mathbf{n}}{\partial y}\right)\\ &= \mathbf{n}\cdot\left(\frac{\partial \mathbf{n}}{\partial x}\times\frac{\partial \mathbf{n}}{\partial y}\right)\end{aligned} \tag{25}$$

Substitute Equation 25 back into Equation 23 and we can get:

$$\begin{aligned}S' &= \frac{1}{4\pi}\iint_\sigma \mathbf{n}'\cdot\left(\frac{\partial \mathbf{n}'}{\partial x}\times\frac{\partial \mathbf{n}'}{\partial y}\right)dxdy\\ &= \frac{1}{4\pi}\iint_\sigma \mathbf{m}_R\mathbf{n}\cdot\left(\frac{\partial \mathbf{m}_R\mathbf{n}}{\partial x}\times\frac{\partial \mathbf{m}_R\mathbf{n}}{\partial y}\right)dxdy\\ &= \frac{1}{4\pi}\iint_\sigma \mathbf{n}\cdot\left(\frac{\partial \mathbf{n}}{\partial x}\times\frac{\partial \mathbf{n}}{\partial y}\right)dxdy\\ &= S\end{aligned} \tag{26}$$

Thus it is proved that the skyrmion number does not change after the beam passes through an arbitrary retarder.

Supplementary Method 4: Comparison between Polar optimization (PO) and Cartesian optimization (CO)

The aim of the section is to find the optimal configuration of different FPUs towards different SoPs (both circular and linear). Note we now name new method 1 as Cartesian optimization (CO), and method 2 as Polar optimization (PO). Proper criteria ($\mathbb{S}$ sharpness; details see below) are extracted from the obtained intensity patterns to evaluate its sensing ability. Note for each FPU, we take advantage of its 'full analysing' properties (which in effect leads to different intensity patterns) towards different input SoPs [6]. Previously, this property of FPU has been harnessed to find the locations of the brightest points within generated intensity patterns to determine the input SoP through an image-based sensing paradigm [6]. Here we focus on image-based analysis as well. Note that we are aware that the FPU realised by the current compound modulator is imperfect. However, the core of this work is to focus the demonstration on showcasing the tunability of the arbitrary retarder array using the traditional calculation method. It does not hinder us from thoroughly evaluating PO and CO in preparation for a further developed and improved platform. Let us start with an arbitrary Stokes vector S on the Poincaré sphere, which can be expressed by two angles $\chi \in [-\frac{\pi}{2}, \frac{\pi}{2}]$ and $\psi \in [-\pi, \pi]$:

$$S = \begin{bmatrix} 1 \\ \cos\chi\cos\psi \\ \cos\chi\sin\psi \\ \sin\chi \end{bmatrix} \tag{27}$$

The Mueller matrix of a Full Poincaré unit (FPU) with fast axis orientation $\theta \in [-\frac{\pi}{2}, \frac{\pi}{2}]$ and retardance value $\delta \in [0, \pi]$ is expressed as [6]:

$$
\begin{aligned}
\mathbf{M}_{\theta,\delta} &= \begin{bmatrix} 1 & 0 & 0 & 0 \\ 0 & \cos 2\theta & -\sin 2\theta & 0 \\ 0 & \sin 2\theta & \cos 2\theta & 0 \\ 0 & 0 & 0 & 1 \end{bmatrix} \begin{bmatrix} 1 & 0 & 0 & 0 \\ 0 & 1 & 0 & 0 \\ 0 & 0 & \cos\delta & \sin\delta \\ 0 & 0 & -\sin\delta & \cos\delta \end{bmatrix} \begin{bmatrix} 1 & 0 & 0 & 0 \\ 0 & \cos 2\theta & \sin 2\theta & 0 \\ 0 & -\sin 2\theta & \cos 2\theta & 0 \\ 0 & 0 & 0 & 1 \end{bmatrix} \\
&= \begin{bmatrix} 1 & 0 & 0 & 0 \\ 0 & \cos^2 2\theta + \sin^2 2\theta\cos\delta & \sin 2\theta\cos 2\theta(1-\cos\delta) & -\sin 2\theta\sin\delta \\ 0 & \sin 2\theta\cos 2\theta(1-\cos\delta) & \sin^2 2\theta + \cos^2 2\theta\cos\delta & \cos 2\theta\sin\delta \\ 0 & \sin 2\theta\sin\delta & -\cos 2\theta\sin\delta & \cos\delta \end{bmatrix}
\end{aligned} \tag{28}
$$

Hence, the light field after a circular polariser can be computed as:

$$
O = \mathbf{P}\mathbf{M}_{\theta,\delta}S = \begin{bmatrix} \frac{1}{2}[1+\cos\delta\sin\chi+\cos\chi\sin\delta\sin(2\theta-\psi)] \\ 0 \\ 0 \\ \frac{1}{2}[1+\cos\delta\sin\chi+\cos\chi\sin\delta\sin(2\theta-\psi)] \end{bmatrix} \tag{29}
$$

where

$$
\mathbf{P} = \frac{1}{2}\begin{bmatrix} 1 & 0 & 0 & 1 \\ 0 & 0 & 0 & 0 \\ 0 & 0 & 0 & 0 \\ 1 & 0 & 0 & 1 \end{bmatrix} \tag{30}
$$

The intensity field measured from the output light can therefore be expressed as:

$$
\mathcal{I}(\theta,\delta) = \frac{1}{2}[1+\cos\delta\sin\chi+\cos\chi\sin\delta\sin(2\theta-\psi)] \tag{31}
$$

We first compute the gradients of the intensity field $\nabla\mathcal{I}(\theta,\delta)$ and the corresponding divergence field $\nabla\cdot\nabla\mathcal{I}(\theta,\delta)$ with respect to θ and δ, respectively. Based on different optimisation strategies, θ and δ can be interpreted as different axes in two coordinate systems.

For Cartesian optimisation (CO), (θ,δ) is interpreted as (x,y) in polar coordinate systems and the corresponding nabla and Laplacian operators are formulated as:

$$
\nabla = \frac{\partial}{\partial\theta}\mathbf{e}_\theta + \frac{\partial}{\partial\delta}\mathbf{e}_\delta \tag{32}
$$

$$
\Delta = \nabla\cdot\nabla = \frac{\partial^2}{\partial\theta^2} + \frac{\partial^2}{\partial\delta^2} \tag{33}
$$

The gradient is computed as:

$$
\begin{aligned}
\frac{\partial\mathcal{I}(\theta,\delta)}{\partial\theta} &= \cos\chi\sin\delta\cos(2\theta-\psi) \\
\frac{\partial\mathcal{I}(\theta,\delta)}{\partial\delta} &= \frac{1}{2}[\sin\delta\sin\chi+\cos\chi\cos\delta\sin(2\theta-\psi)]
\end{aligned} \tag{34}
$$

And the divergence field of the gradient is expressed as:

$$
\begin{aligned}
\Delta\mathcal{I}(\theta,\delta) &= \frac{\partial^2\mathcal{I}}{\partial\theta^2} + \frac{\partial^2\mathcal{I}}{\partial\delta^2} \\
&= -\frac{1}{2}[\cos\chi\sin\delta\sin(2\theta-\psi)+\cos\delta\sin\chi]
\end{aligned} \tag{35}
$$

In Polar optimisation (PO), (θ,δ) is interpreted as (ϕ,r) in polar coordinate systems, and the corresponding nabla and Laplacian operators are formulated as:

$$
\nabla = \frac{\partial}{\partial\delta}\mathbf{e}_\delta + \frac{1}{\delta}\frac{\partial}{\partial\theta}\mathbf{e}_\theta \tag{36}
$$

$$
\Delta = \nabla\cdot\nabla = \frac{1}{\delta}\frac{\partial}{\partial\delta}\left(\delta\frac{\partial}{\partial\delta}\right) + \frac{1}{\delta^2}\frac{\partial^2}{\partial\theta^2} \tag{37}
$$

The gradient is computed as:

$$
\begin{aligned}
\frac{1}{\delta}\frac{\partial \mathcal{I}(\theta,\delta)}{\partial \theta} &= \frac{1}{\delta}\cos\chi\sin\delta\cos(2\theta-\psi) \\
\frac{\partial \mathcal{I}(\theta,\delta)}{\partial \delta} &= \frac{1}{2}[\sin\delta\sin\chi+\cos\chi\cos\delta\sin(2\theta-\psi)]
\end{aligned}
\tag{38}
$$

And the divergence field of the gradient is expressed as:

$$
\begin{aligned}
\Delta\mathcal{I}(\theta,\delta) &= \frac{1}{\delta}\frac{\partial \mathcal{I}}{\partial \delta}\left(\delta\frac{\partial \mathcal{I}}{\partial \delta}\right)+\frac{1}{\delta^2}\frac{\partial^2 \mathcal{I}}{\partial \theta^2} \\
&= -\frac{1}{2\delta^2}\left[\delta\sin\delta\sin\chi-\delta\cos\chi\cos\delta\sin(2\theta-\psi)+\cdots\right. \\
&\delta^2\cos\delta\sin\chi+\delta^2\cos\chi\sin\delta\sin(2\theta-\psi)+\cdots \\
&\left.2\cos\chi\sin\delta\sin(2\theta-\psi)\right]
\end{aligned}
\tag{39}
$$

For this work, to retrieve the input SoP through physical inference, the maximum point and minimum point are both located to provide the necessary information that determines the SoP. Based on the extreme value theorem, the maximizer and minimizer of $\mathcal{I}(\theta,\delta)$ can be obtained from the local extremes within the domain, or on the boundary [6].

Boundary. There are four boundaries in the variable domain:

$$
\begin{aligned}
\mathcal{B}_1 &= \{\theta\in[-\frac{\pi}{2},\frac{\pi}{2}],\delta=0\} \\
\mathcal{B}_2 &= \{\theta\in[-\frac{\pi}{2},\frac{\pi}{2}],\delta=\pi\} \\
\mathcal{B}_3 &= \{\theta=-\frac{\pi}{2},\delta\in[0,\pi]\} \\
\mathcal{B}_4 &= \{\theta=\frac{\pi}{2},\delta\in[0,\pi]\}
\end{aligned}
$$

And the evaluation of $\mathcal{I}(\theta,\delta)$ on these boundaries is computed as follows:

$$
\begin{aligned}
\mathcal{B}_1:\mathcal{I}(\theta,\delta) &= \frac{1}{2}(1+\sin\chi) \\
\mathcal{B}_2:\mathcal{I}(\theta,\delta) &= \frac{1}{2}(1-\sin\chi) \\
\mathcal{B}_3:\mathcal{I}(\theta,\delta) &= \frac{1}{2}(1+\cos\delta\sin\chi+\cos\chi\sin\delta\sin\psi) \\
\mathcal{B}_4:\mathcal{I}(\theta,\delta) &= \frac{1}{2}(1+\cos\delta\sin\chi+\cos\chi\sin\delta\sin\psi)
\end{aligned}
$$

Therefore, the extremes on the boundaries are obtained at $(\theta=\frac{\pi}{2},\delta=\delta_b)$ and $(\theta=-\frac{\pi}{2},\delta=\delta_b)$, since the evaluation of $\mathcal{B}_3$, $\mathcal{B}_4$ incorporate the cases in $\mathcal{B}_1$, $\mathcal{B}_2$. If $\delta_b\neq 0,\pi$, then $\left.\frac{\partial\mathcal{I}(\theta,\delta)}{\partial\theta}\right|_{\theta=-\frac{\pi}{2}} = -\cos\chi\cos\psi\sin\delta_b = \left.\frac{\partial\mathcal{I}(\theta,\delta)}{\partial\theta}\right|_{\theta=\frac{\pi}{2}}$, suggesting that the maximizer and minimizer are obtained from local extremes.

Local extremes. Next, we consider the local extremes. After making Equation 34 and Equation 38 equal to zero, we can find that in both CO and PO, the following expressions hold:

$$
\begin{aligned}
\cos(2\theta-\psi)=0 &\Rightarrow 2\theta-\psi=-\frac{3}{2}\pi,-\frac{1}{2}\pi,\frac{1}{2}\pi,\frac{3}{2}\pi \\
\sin\delta\sin\chi+\cos\chi\cos\delta\sin(2\theta-\psi)=0 &\Rightarrow \cos(\delta\pm\chi)=0
\end{aligned}
\tag{40}
$$

By analyzing the local maximizer and minimizer based on the equation above, we can find that θ and δ are one-to-one functions of (χ,ψ). For maximizer:

$$
\begin{aligned}
\cos(2\theta|_{\text{maximizer}}-\psi) &= 0 \\
\sin(2\theta|_{\text{maximizer}}-\psi) &= 1 \\
\delta|_{\text{maximizer}} &= \frac{\pi}{2}-\chi
\end{aligned}
\tag{41}
$$

And for minimizer:

$$\begin{aligned}\cos(2\theta|_{\text{minimizer}} - \psi) = 0\\ \sin(2\theta|_{\text{minimizer}} - \psi) = -1\\ \delta|_{\text{minimizer}} = \frac{\pi}{2} + \chi\end{aligned} \tag{42}$$

To retrieve the input SoP from the captured image through physical inference, the maximum point and minimum point of the intensity profile should be located precisely. Considering the pixelated nature of the intensity sensor (e.g. CMOS camera etc.), the sharper the extreme points, the more accurately their positions can be located by the sensor. In order to quantitatively evaluate the accuracy and quality of the retrieval process, we analyse the divergence field of the intensity gradient. For one extreme point (the brightest point), the absolute magnitude of the divergence should be the highest, which can be distinguished by a significant decay of the intensity around the extreme point. And for the other extreme point (darkest point), the divergence should be the lowest.

So here, we define a new evaluation coefficient sharpness $\mathbb{S}$, by considering the sum of the absolute divergence at both maximum and minimum points for PO and CO. It is expressed as:

$$\mathbb{S} = |\Delta\mathcal{I}|_{@\max}| + |\Delta\mathcal{I}|_{@\min}| \tag{43}$$

For CO,

$$\mathcal{I}_{\text{CO}}|_{@\max} = -2\cos^2\chi - \frac{1}{2} \tag{44}$$

$$\mathcal{I}_{\text{CO}}|_{@\min} = 2\cos^2\chi + \frac{1}{2} \tag{45}$$

thus,

$$\mathbb{S}_{\text{CO}} = 4\cos^2\chi + 1 \tag{46}$$

For PO,

$$\mathcal{I}_{\text{PO}}|_{@\max} = -\frac{2}{(\frac{\pi}{2} - \chi)^2}\cos^2\chi - \frac{1}{2} \tag{47}$$

$$\mathcal{I}_{\text{PO}}|_{@\min} = \frac{2}{(\frac{\pi}{2} + \chi)^2}\cos^2\chi + \frac{1}{2} \tag{48}$$

thus,

$$\mathbb{S}_{\text{PO}} = \left(\frac{2}{(\frac{\pi}{2} - \chi)^2} + \frac{2}{(\frac{\pi}{2} + \chi)^2}\right)\cos^2\chi + 1 \tag{49}$$

We can find that the quality of the measurement is only related to the ellipticity χ of the incoming SoP. By analysing the sharpness of PO and CO with their extreme values, we can find some unique properties for PO and CO. For the sharpness of CO, it has a maximum value when $\chi = 0$ and two minimum values when $\chi = \pm\frac{\pi}{2}$ and is monotonic between the extremes. Based on the definition of sharpness we proposed before, we can conclude that CO can measure linear SoPs more accurately compared with circular SoPs when using pixelated intensity sensors. And for the sharpness of PO, it has a minimum value when $\chi = 0$ and two maximum values when $\chi = \pm\frac{\pi}{2}$ and is monotonic between the extremes. We can conclude that PO can measure circular SoPs more accurately compared to linear SoPs when using pixelated intensity sensors.

In summary, PO and CO are proposed and an evaluation coefficient sharpness $\mathbb{S}$ is proposed to evaluate the SoP retrieving process using these two optimisation procedures.

Supplementary Method 5: Object-wise adaptive optics (O-AO)

Sensorbased method

This method contains two core parts. The first part is the initial calibration and characterization of the SLMs, whose dynamic range will need to be measured and used in the following experiments. A LUT will be generated after the calibration, preparing for the correction process [7]. The second part is to use the Mueller matrix polarimeter to directly measure the polarisation aberration introduced by an external target in a pixelated way. The phase patterns on the SLMs will be adjusted based on the LUT to compensate for the measured polarization aberration.

The residual phase aberration will then be corrected by the deformable mirror (DM). Both the polarization and phase aberrations are expected to be corrected after the sensor-based method.

To generate the LUT for the SLMs modulation, the following steps are taken. Initially, the source laser intensity is adjusted using a neutral-density (ND) filter, and the exposure time of the CCD camera is carefully chosen to ensure the calibration data will use the full dynamic range of the CCD camera. These parameters are fixed throughout the experiment. Each SLM pixel has an 8-bit modulation depth, with flat grayscale values F_i ranging from 0 to 255 ($F_i \in [0, 255]$, $F_i \in \mathbb{Z}$). Each flat value F_i is applied across the SLM, and the corresponding intensity images are captured. Because the size of the captured image is larger than the pattern that is loaded onto the SLM, the image will then be resized to fit the number of pixels on the SLM using the bicubic method. Thus, the corresponding intensities with all N pixels assigned with the flat value F_i will be derived and converted back to the phase introduced as $[P_{i_1}, P_{i_2}, \ldots, P_{i_N}]$, where P_{i_n} stands for the corresponding phase value of nth pixel applied flat value F_i. This process is iterated for all grayscale values F_i, resulting in a dynamic range of phase values $[P_{0_n}, P_{1_n}, \ldots, P_{255_n}]$ for each pixel, typically forming a trigonometric function. The result is then unwrapped based on the minimum phase value S_n and the maximum phase value L_n at the corresponding flat value F_S and F_L. After these processes, the resulting linear mapping between input flat values F_{in} and the output phase values P_{out} is saved into the LUT for each SLM for further utilization.

For the correction process, all SLMs in the system are flattened (using the LUT generated before to have a uniform phase profile), and images of the SLM surface and the focal intensity distribution (FID) of the system are recorded as references. After that, the DM sensorless correction process is executed to calibrate and compensate for the system's phase aberration. A standard Airy disk shape will be recorded to indicate that the system has been calibrated. After this, an external vectorial aberration is introduced into the system. By utilizing Mueller matrix polarimetry, the polarisation aberration D introduced by the external sample can be quantitatively obtained without any iterations. To correct the aberration, the explementary value of the polarisation aberration valued as $2\pi - D$ will be decomposed into three individual modes D_1, D_2, D_3. These modes will be applied to the three SLMs to form an "explementary elliptical retarder" to compensate for the aberration (implementation refer to Supplementary Method 1). After the O-AO sensor-based method, a post-DM sensorless correction is followed to further improve the FID quality [8, 9, 10]. The related flow chart can be found in Supplementary Note 4.

Sensorless method

The core idea of this sensor-less method is to iteratively apply a group of designed retardance patterns onto SLMs to compensate for the vectorial aberration. Here, only the FID images will be captured and a selection of designed patterns for O-AO correction will be scanned across a given pattern coefficient range to obtain the optimal pattern for aberration correction. The SLMs are assumed to be calibrated as described in the previous section.

For the correction process, all SLMs in the system are initially flattened and images of the system FID are captured as reference. Then, the same calibration process described in the previous section is conducted. Afterwards, the same external aberration is again introduced into the system, with the same assumption as that in vectorial adaptive optics [7] (i.e., the axis orientation is known as prior knowledge). In the O-AO sensorless approach, a group of novel retardance patterns (which we term vectorial retardance modes (see Supplementary Note 5)) are applied to our synthetic retarder array during the aberration correction procedure. The modes utilized in this method have controllable parameters similar to standard Zernike modes, with the exception that the phase values are replaced by the retardance values. The real-valued retardance aberration function $\delta(\rho, \theta)$, which is defined over the unit disk, is given by [11]

$$\delta(\rho, \theta) = \sum_{n,m} \alpha_n^m \mathcal{Z}_n^m(\rho, \theta) \tag{50}$$

where the definition of n and m follows the one given by Zernike [11] and Noll [12]. $\alpha_n^m \in \mathbb{R}$ are the coefficients of the real-valued Zernike polynomials $\mathcal{Z}_n^m$, which are defined by [12, 13]

$$\mathcal{Z}_n^m(\rho, \theta) = c_n^m R_n^{|m|}(\rho) \Theta_n^m(\theta) \tag{51}$$

where

$$c_n^m = \begin{cases} \sqrt{n+1} & m = 0 \\ \sqrt{2(n+1)} & m \neq 0 \end{cases} \tag{52}$$

$$R_n^{|m|}(\rho) = \sum_{s=0}^{(n-|m|)/2} \frac{(-1)^s (n-s)!}{s![(n+|m|)/2 - s]![(n-|m|)/2 - s]!} \rho^{n-2s} \tag{53}$$

$$\Theta_n^m(\theta) = \begin{cases} \cos(m\theta) & m \geqslant 0 \\ -\sin(m\theta) & m < 0 \end{cases} \tag{54}$$

A series of vectorial retardance modes from $\mathcal{Z}_1$ to $\mathcal{Z}_{20}$ (according to Noll's indexing convention) are applied to the SLMs by splitting the retardance pattern into three sub-modes ϕ_1, ϕ_2 ϕ_3, and applying them on SLMs. Before the recording of each mode pattern, the optimal α_k needs to be determined referring to FID images by interating it from -1.0 to 1.0 in steps of 0.1. After the O-AO sensorless method, a post-DM sensorless phase AO correction follows to further improve the focus quality by eliminating residual phase errors. See the corresponding flow chart in Supplementary Note 4.

It is worth noting that the correction accuracy may be reduced when the deviation is complex or exceeds the available mode range, leading to slower convergence and increased iterations. Additionally, the correction quality may degrade under strong superposition of mode patterns due to the SLM pixel resolution, particularly for large deviations or high-frequency aberrations. However, these limitations are highly likely to be mitigated by refining the calibration process for mode superposition with more modes using higher-resolution SLMs. Furthermore, expanding the mode range with enhanced learning-based correction algorithms [21] could further enhance accuracy and efficiency.

Supplementary Method 6: Pixelated control of the intensity value

The SLM we used consisted of arrays of pixelated nematic liquid crystal (LC) units, which can be used for pixelated intensity control (see Supplementary Note 5). By putting a pair of linear polarisers before (P1) and after (P2) the SLM, pixelated control of the intensity value can be quantitatively achieved by controlling the SLM settings. This method has been used in modern optics, while here we generalise it into our cascaded structure matter regime. Note theoretically other low functionality devices may also serve this purpose, such as pixelated attenuator arrays, although the mathematical description would be then different.

Suppose that the wave equation of the incident beam after the incident vertical linear polariser is written as:

$$\mathbf{E}_\mathrm{i} = E_y \cos(wt)\hat{\mathbf{y}} \tag{55}$$

If P2 is parallel to P1, then the electric field after P2 can be simplified as:

$$\mathbf{E}_\mathrm{o} = E_y \left[\cos\left(wt - \frac{\pi n_m}{\lambda} d\right)\cos\left(\frac{\pi \Delta n}{\lambda} d\right) + \cos(2\chi)\sin\left(wt - \frac{\pi n_m}{\lambda} d\right)\sin\left(\frac{\pi \Delta n}{\lambda} d\right)\right]\hat{\mathbf{x}} \tag{56}$$

where $\Delta n = n_e - n_o$ is the birefringence of liquid crystals and $n_m = n_e + n_o$.

When the angle between the polariser and LC director χ is exactly 45°, then the whole expression can be simplified to:

$$\mathbf{E}_\mathrm{o} = E_y \cos\left(\frac{\pi \Delta n}{\lambda} d\right)\cos\left(wt - \frac{\pi n_m}{\lambda} d\right)\hat{\mathbf{x}} \tag{57}$$

So the transmission rate under this situation can be expressed by the ratio between the two intensities $\frac{\mathrm{I_o}}{\mathrm{I_i}}$ as

$$\begin{aligned} T &= \cos^2\left(\frac{\pi \Delta n d}{\lambda}\right) \quad \text{when } \chi = 45^\circ \\ &= \frac{1}{2} + \frac{1}{2}\cos\left(\frac{2\pi \Delta n d}{\lambda}\right) \quad \text{when } \chi = 45^\circ \end{aligned} \tag{58}$$

Supplementary Method 7: Pixelated control of the degree of polarisation (DoP)

Depolarisation represents the reduction in the DoP and is related to incoherent spatiotemporal coupling of polarised light into unpolarised light [3]. In this section, we harness another configuration of the cascaded devices (synthetic matter; see Supplementary Note 5) to achieve pixelated control of the depolarisation patterns by applying time-dependent retardance patterns.

The strategy for realizing pixelated control of DoP is to use cascaded devices to generate pixelated time-varying retardance patterns, resulting in a temporally varying SoP in each pixel. This will introduce a depolarisation effect if the temporal variation is faster than the integration time of the detector [14, 15].

A generated temporally varying SoP can be described as an incoherent superposition of n Stokes vectors that represent n fully polarised SoPs. The effective Stokes vector of the temporally varying SoP can be expressed by averaging the n SoPs within a time period T

$$\langle S\rangle = \frac{1}{T}[t_1S_1 + t_2S_2 + \cdots + t_nS_n] \tag{59}$$

where $t_1 + t_2 + \cdots + t_n = T$

To achieve pixelated depolarisation modulation, n phase patterns with different retardance values are consecutively applied to the synthetic matter during the integration time of the detector. For a homogeneous input SoP, each retardance pattern can be described by a Mueller matrix M_n of the matter:

$$\begin{aligned} S_1(x,y) &= M_1(x,y)S_{\text{in}}(x,y) \\ &\vdots \\ S_n(x,y) &= M_n(x,y)S_{\text{in}}(x,y) \end{aligned} \tag{60}$$

where (x, y) represents the spatial coordinate of the matter. The device we use in this section is the cascade of SLMs, whose Mueller matrix can be expressed as:

$$M = M_{\text{Z}}\cdots M_{\text{B}}M_{\text{A}} \tag{61}$$

For a combination of 3 SLMs with their fast axis arranged in a sequence of 0°, 45° , and 0°, the corresponding retardance profile ϕ_{A}, ϕ_{B}, ϕ_{C} will lead to the Mueller matrix being in the form of:

$$M = \begin{bmatrix} 1 & 0 & 0 & 0 \\ 0 & \cos\phi_{\text{B}} & 0 & -\sin\phi_{\text{B}} \\ 0 & \sin(\phi_{\text{A}}+\phi_{\text{C}})\sin\phi_{\text{B}} & \cos(\phi_{\text{A}}+\phi_{\text{C}}) & \sin(\phi_{\text{A}}+\phi_{\text{C}})\cos\phi_{\text{B}} \\ 0 & \cos(\phi_{\text{A}}+\phi_{\text{C}})\sin\phi_{\text{B}} & -\sin(\phi_{\text{A}}+\phi_{\text{C}}) & \cos(\phi_{\text{A}}+\phi_{\text{C}})\cos\phi_{\text{B}} \end{bmatrix} \tag{62}$$

The Mueller matrix of depolarisation modulation effects during the integration period T can be expressed as:

$$\langle M(x,y)\rangle = \frac{1}{T}[t_1M_1(x,y) + t_2M_2(x,y) + \cdots + t_nM_n(x,y)] \tag{63}$$

where M_i represents the different combinations of ϕ_{A}, ϕ_{B}, ϕ_{C}.

For the initial demonstration, we assume that the number of retardance patterns $n = 2$ and temporal variation $t_1 = t_2 = \frac{T}{2}$. And also we first assume that only SLM B will change its retardance in this demonstration. Hence the Mueller matrix of the depolarisation modulation effect during integration period T can be described as:

$$\langle M(x,y)\rangle = \frac{1}{2}[M_1(x,y) + M_2(x,y)] \tag{64}$$

where M_1 has the retardance combination of ϕ_{A}, ϕ_{B_1}, ϕ_{C} and M_2 has the retardance combination of ϕ_{A}, ϕ_{B_2}, ϕ_{C}.

For an arbitrarily illuminated fully polarised SoP $S_{\text{in}} = [S_0, S_1, S_2, S_3]^T$, the effective output Stokes vector can be obtained:

$$S_{\text{out}} = \frac{1}{2}\begin{bmatrix} 2S_0 \\ (\cos\phi_{\text{B}_1}+\cos\phi_{\text{B}_2})S_1 - (\sin\phi_{\text{B}_1}+\sin\phi_{\text{B}_2})S_3 \\ \sin(\phi_{\text{A}}+\phi_{\text{C}})\left[(\sin\phi_{\text{B}_1}+\sin\phi_{\text{B}_2})S_1 + (\cos\phi_{\text{B}_1}+\cos\phi_{\text{B}_2})S_3\right] + 2\cos(\phi_{\text{A}}+\phi_{\text{C}})S_2 \\ \cos(\phi_{\text{A}}+\phi_{\text{C}})\left[(\sin\phi_{\text{B}_1}+\sin\phi_{\text{B}_2})S_1 + (\cos\phi_{\text{B}_1}+\cos\phi_{\text{B}_2})S_3\right] - 2\sin(\phi_{\text{A}}+\phi_{\text{C}})S_2 \end{bmatrix} \tag{65}$$

Hence the DoP is expressed as:

$$\begin{aligned} \text{DoP} &= \sqrt{\frac{{S_{\text{out}}}_1^2 + {S_{\text{out}}}_2^2 + {S_{\text{out}}}_3^2}{{S_{\text{out}}}_0^2}} \\ &= \sqrt{\frac{S_0^2 + \cos(\phi_{\text{B}_1}-\phi_{\text{B}_2})S_1^2 + S_2^2 + \cos(\phi_{\text{B}_1}-\phi_{\text{B}_2})S_3^2}{2S_0^2}} \\ &= \sqrt{\left(\frac{S_2}{S_0}\right)^2 \sin^2\left(\frac{\phi_{\text{B}_1}-\phi_{\text{B}_2}}{2}\right) + \cos^2\left(\frac{\phi_{\text{B}_1}-\phi_{\text{B}_2}}{2}\right)} \end{aligned} \tag{66}$$

For a uniform input polarisation field (say S_2 equals to 0 across all the pixels), any pixelated output DoP level can be achieved by manipulating the retardance patterns. Hence, arbitrary DoPs can be manipulated in a pixelated manner via synthetic matter.

Note here we also for the first time put forward the concept of DoP control via a 3 SLM geometry, in which we can adopt an arbitrary non-uniform SoP field as input, via modulating SLM A and SLM C together with SLM B. Conceptually, one can understand that 3 SLMs hold the ability of conversion between arbitrary SoPs (also can refer to Supplementary Method 1), thus for any input SoP, we can make the synthetic matter generate expected SoPs via time. This will lead to the pixelated generation of any depolarisation levels for any input SoP.

Supplementary Method 8: Complex cascades to form arbitrary diattenuators

In this section, we give mathematical proof of one possible structure that can form an arbitrary diattenuator (with infinite extinction ratio) via combinations of retarders and a fixed polariser (see Supplementary Note 5). Note here we first focus on a perfect polariser. The cascaded model can be expressed as:

$$\mathbf{M}_{\mathrm{AD_A}} = \mathbf{M}_{\mathrm{AR_1}} \cdot \mathbf{P} \cdot \mathbf{M}_{\mathrm{AR_2}} \tag{67}$$

where $\mathbf{AD}_{\mathrm{A}}$ is the Mueller matrix of the arbitrary diattenuator that we want to achieve, $\mathbf{M}_{\mathrm{AR_1}}$ and $\mathbf{M}_{\mathrm{AR_2}}$ are the Mueller matrices of the arbitrary retarders before and after a certain diattenuator and $\mathbf{P}$ is the Mueller matrix of the fixed polariser with an arbitrary axis.

From the Jones matrix expression of the arbitrary retarder

$$\mathbf{J}_{\mathrm{AR}} = \begin{bmatrix} \cos\alpha & -\sin\alpha e^{-i\delta} \\ \sin\alpha e^{i\delta} & \cos\alpha \end{bmatrix} \begin{bmatrix} e^{i\psi+i\phi/2} & 0 \\ 0 & e^{i\psi-i\phi/2} \end{bmatrix} \begin{bmatrix} \cos\alpha & \sin\alpha e^{-i\delta} \\ -\sin\alpha e^{i\delta} & \cos\alpha \end{bmatrix} \tag{68}$$

its Mueller matrix can be expressed as:

$$\begin{aligned} \mathbf{M}_{\mathrm{AR}} &= \begin{bmatrix} 1 & 0 & 0 & 0 \\ 0 & s_1^2(1-\cos\phi)+\cos\phi & s_1 s_2(1-\cos\phi)+s_3\sin\phi & s_1 s_3(1-\cos\phi)-s_2\sin\phi \\ 0 & s_1 s_2(1-\cos\phi)-s_3\sin\phi & s_2^2(1-\cos\phi)+\cos\phi & s_2 s_3(1-\cos\phi)+s_1\sin\phi \\ 0 & s_1 s_3(1-\cos\phi)+s_2\sin\phi & s_2 s_3(1-\cos\phi)-s_1\sin\phi & s_3^2(1-\cos\phi)+\cos\phi \end{bmatrix} \\ &= \begin{bmatrix} 1 & \mathbf{0} \\ \mathbf{0}^T & \mathbf{m}_R \end{bmatrix} \end{aligned} \tag{69}$$

where

$$S = \begin{bmatrix} s_0 \\ s_1 \\ s_2 \\ s_3 \end{bmatrix} = \begin{bmatrix} 1 \\ \cos 2\alpha \\ \sin 2\alpha \cos\delta \\ \sin 2\alpha \sin\delta \end{bmatrix} \tag{70}$$

is the Stokes vector form of the eigenvector of the arbitrary retarder and $\mathbf{m}_R$ is the orthogonal rotation matrix.

From the Jones matrix expression, a polariser with a fixed axis can be expressed as:

$$\mathbf{J}_{\mathrm{AD}} = \begin{bmatrix} \cos\alpha & -\sin\alpha e^{-i\delta} \\ \sin\alpha e^{i\delta} & \cos\alpha \end{bmatrix} \begin{bmatrix} \cos\gamma & 0 \\ 0 & \sin\gamma \end{bmatrix} \begin{bmatrix} \cos\alpha & \sin\alpha e^{-i\delta} \\ -\sin\alpha e^{i\delta} & \cos\alpha \end{bmatrix} \tag{71}$$

and its Mueller matrix can be expressed as:

$$\mathbf{M}_{\mathrm{AD}} = \frac{1}{2} \begin{bmatrix} 1 & \mathbf{D}^{\mathrm{T}} \\ \mathbf{D} & \mathbf{m}_D \end{bmatrix} \tag{72}$$

where

$$\begin{aligned} \mathbf{m}_D &= \begin{bmatrix} \sin 2\gamma + (1-\sin 2\gamma)s_1^2 & (1-\sin 2\gamma)s_1 s_2 & (1-\sin 2\gamma)s_1 s_3 \\ (1-\sin 2\gamma)s_1 s_2 & \sin 2\gamma + (1-\sin 2\gamma)s_2^2 & (1-\sin 2\gamma)s_2 s_3 \\ (1-\sin 2\gamma)s_1 s_3 & (1-\sin 2\gamma)s_2 s_3 & \sin 2\gamma + (1-\sin 2\gamma)s_3^2 \end{bmatrix} \\ &= \sin 2\gamma \mathbf{I}_{3\times 3} + (1-\sin 2\gamma)\mathbf{S}\cdot\mathbf{S}^T \end{aligned} \tag{73}$$

$$\mathbf{D} = \cos 2\gamma \begin{bmatrix} s_1 \\ s_2 \\ s_3 \end{bmatrix} = \cos 2\gamma \mathbf{S} \tag{74}$$

with the definition of

$$S = \begin{bmatrix} s_0 \\ s_1 \\ s_2 \\ s_3 \end{bmatrix} = \begin{bmatrix} 1 \\ \cos 2\alpha \\ \sin 2\alpha \cos \delta \\ \sin 2\alpha \sin \delta \end{bmatrix} = \begin{bmatrix} 1 \\ \mathbf{S} \end{bmatrix} \tag{75}$$

So the final cascaded structure can be expressed as:

$$\begin{aligned} \mathbf{M}_{\mathrm{AR}_1} \cdot \mathbf{P} \cdot \mathbf{M}_{\mathrm{AR}_2} &= \frac{1}{2} \begin{bmatrix} 1 & \mathbf{0} \\ \mathbf{0}^T & \mathbf{m}_{R_1} \end{bmatrix} \begin{bmatrix} 1 & \mathbf{D}^{\mathrm{T}} \\ \mathbf{D} & \mathbf{m}_D \end{bmatrix} \begin{bmatrix} 1 & \mathbf{0} \\ \mathbf{0}^T & \mathbf{m}_{R_2} \end{bmatrix} \\ &= \frac{1}{2} \begin{bmatrix} 1 & \mathbf{D}^{\mathrm{T}} \mathbf{m}_{R_2} \\ \mathbf{m}_{R_1} \mathbf{D} & \mathbf{m}_{R_1} \mathbf{m}_D \mathbf{m}_{R_2} \end{bmatrix} \end{aligned} \tag{76}$$

To form an arbitrary diattenuator $\mathbf{M}_{\mathrm{AD}_\mathrm{A}}$ using this cascaded structure, the following equations should hold:

$$\mathbf{D}^{\mathrm{T}} \mathbf{m}_{R_2} = \mathbf{D}_{\mathrm{A}}^{\mathrm{T}} \tag{77}$$

$$\mathbf{m}_{R_1} \mathbf{D} = \mathbf{D}_{\mathrm{A}} \tag{78}$$

$$\mathbf{m}_{R_1} \mathbf{m}_D \mathbf{m}_{R_2} = \mathbf{m}_{D_\mathrm{A}} \tag{79}$$

From Equation 77 and Equation 78, considering that $\mathbf{m}_{R_1}$ and $\mathbf{m}_{R_2}$ are rotation matrices, we find that:

$$\mathbf{m}_{R_1} \cdot \mathbf{m}_{R_2} = \mathbf{I}_{3\times 3} \tag{80}$$

By expanding Equation 79 and assuming that distinction ratio γ is the same for the fixed polariser and resulting arbitrary diattenuator, we obtain:

$$\begin{aligned} \mathbf{m}_{R_1} \mathbf{m}_D \mathbf{m}_{R_2} &= \mathbf{m}_{R_1} (\sin 2\gamma \mathbf{I}_{3\times 3} + (1 - \sin 2\gamma) \mathbf{S} \cdot \mathbf{S}^T) \mathbf{m}_{R_2} \\ &= \sin 2\gamma \mathbf{m}_{R_1} \mathbf{I}_{3\times 3} \mathbf{m}_{R_2} + (1 - \sin 2\gamma) \mathbf{m}_{R_1} \mathbf{S} \cdot \mathbf{S}^T \mathbf{m}_{R_2} \\ &= \sin 2\gamma \mathbf{I}_{3\times 3} + \frac{1 - \sin 2\gamma}{\cos^2 2\gamma} \mathbf{m}_{R_1} \mathbf{D} \cdot \mathbf{D}^T \mathbf{m}_{R_2} \\ &= \sin 2\gamma \mathbf{I}_{3\times 3} + \frac{1 - \sin 2\gamma}{\cos^2 2\gamma} \mathbf{D}_{\mathrm{A}} \cdot \mathbf{D}_{\mathrm{A}}^{\mathrm{T}} \\ &= \sin 2\gamma \mathbf{I}_{3\times 3} + (1 - \sin 2\gamma) \mathbf{S}_{\mathrm{A}} \cdot \mathbf{S}_{\mathrm{A}}^{\mathrm{T}} \\ &= \mathbf{m}_{D_\mathrm{A}} \end{aligned} \tag{81}$$

Thus Equation 77, Equation 78 and Equation 79 hold and they prove that an arbitrary diattenuator (with infinite extinction ratio) can be achieved by cascading several retarders and a fixed polariser.

Supplementary Note 1

A tuneable arbitrary retarder array, consisting of three SLMs and one DM (see Supplementary Figure 1a), is calibrated prior to any applications. Here, we demonstrate the performance of the array before and after calibration quantitatively, assessed through the polarization and phase profiles of the devices. The details of the calibration process can be found in Supplementary Method 2.

The output Stokes vector fields before and after calibration are shown in Supplementary Figure 1. Before calibration, the SLM pixels produce a disordered output field, but after calibration, precise control yields a well-ordered, uniform field. To evaluate the performance of calibration procedure, we introduce two criteria: vectorial precision and vectorial uniformity. We first quantify the vector difference D on the Poincaré sphere between the target state of polarisation (SoP) $S = [S_1, S_2, S_3]$ and the experimentally obtained SoP $S = [\tilde{S}_1, \tilde{S}_2, \tilde{S}_3]$ as:

$$D = c_p \|S - \tilde{S}\|$$

where both S and $\tilde{S}$ are unit vectors ($[-1, 1]$ range for each component) and c_p is a constant normalization factor chosen to keep D within $[0, 1]$. Since $\|S - \tilde{S}\|$ can vary within the range of $[0, 2]$, we set $c_p = \frac{1}{2}$.

We then define the precision metric P:

$$P = 1 - D$$

such that $P = 1$ signifies perfect matching between the target and actual SoP, while $P = 0$ represents the maximum mismatch on the Poincaré sphere.

The uniformity difference U_D is defined as the standard deviation of the actual SoP ($\tilde{S}$) from the mean SoP ($\bar{S}$) across the whole region of interest:

$$U_D = c_u \sqrt{\overline{\|\bar{S} - \tilde{S}\|^2}}$$

where c_u is the constant normalization factor set to 1 to ensure U_D falls within $[0, 1]$. A smaller U_D represents a more uniform distribution. Therefore, we define the uniformity metric U so that larger values of U indicate more uniform SoP distribution:

$$U = 1 - U_D$$

Further quantitative analysis of the output Stokes vector fields demonstrates significant improvements, with vectorial above 95% and uniformity rising from 40.3% to 95.6% (see Supplementary Figure 1c). This demonstrates the effectiveness of calibration in achieving accurate polarisation control. Additionally, the phase pattern on DM to ensure a flat phase profile after calibration is also provided in Supplementary Figure 1b, which is crucial for reducing aberrations and enhancing system performance.

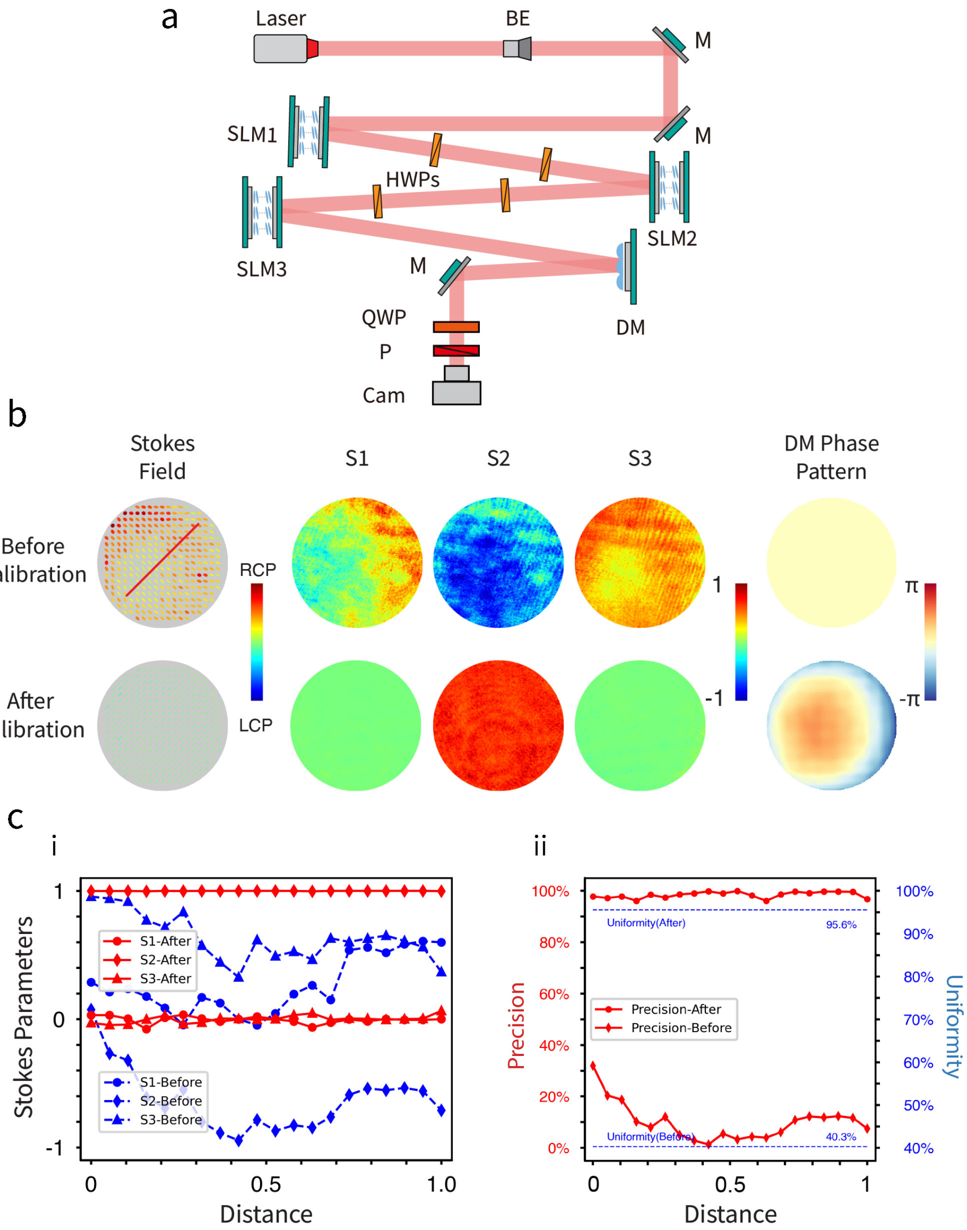

Supplementary Figure 1: Calibration of the tuneable arbitrary retarder array. (**a**) Schematic of the experimental setup. The system consists of a He-Ne laser (Melles Griot, 05-LHP-171, 632.8 nm) as the light source, followed by a beam expander (BE). The tuneable arbitrary retarder array is composed of three SLMs (SLM1, SLM2, SLM3; Hamamatsu, X10468-01) and a DM (Boston Micromachines Corporation, Multi-3.5). Half-wave plates (HWPs; Thorlabs, WPH05M-633) are placed between the SLMs to introduce a 45° relative rotation. A rotating quarter-wave plate (QWP; Thorlabs, WPQ10M-633) and a fixed polarizer (P; Thorlabs, GL10-A) are used for polarimetric measurements, and the output is recorded by a camera (Cam; Thorlabs, DCC3240N). (**b**) Stokes vector fields, individual Stokes parameters and DM phase patterns before and after calibration of the SLMs and DM (see Supplementary Method 2) are given. Different ellipses and colours represent different Stokes vectors, as explained in the main article. To enhance visual clarity, the phase profile on the DM is illustrated and the Stokes ellipses are plotted based on down-sampled measurement data averaged over neighbouring 10×10 pixels. (**c**) (i) Variation in each Stokes parameter along the red line in (c), before and after SLM and DM calibration. Here, the horizontal axis represents sampling positions (pixel indices). (ii) Calculated precision along the same line and uniformity values of the whole plane before and after SLM and DM calibration (see Supplementary Method 2).

Supplementary Note 2

The proof-of-concept results in the main article validated the tuneable arbitrary retarder array as a skyrmionic beam generator. Here the topological protection property of the beams under typical thin media is experimentally validated, with the mathematical proof detailed in Supplementary Method 3.

Topological protection for both beam types is demonstrated under isotropic and anisotropic perturbations through a beam probing system (Supplementary Figure 2a). The consistent skyrmion numbers across both scenarios (Supplementary Figure 2b) illustrated the topological robustness and stability of the skyrmionic beams.

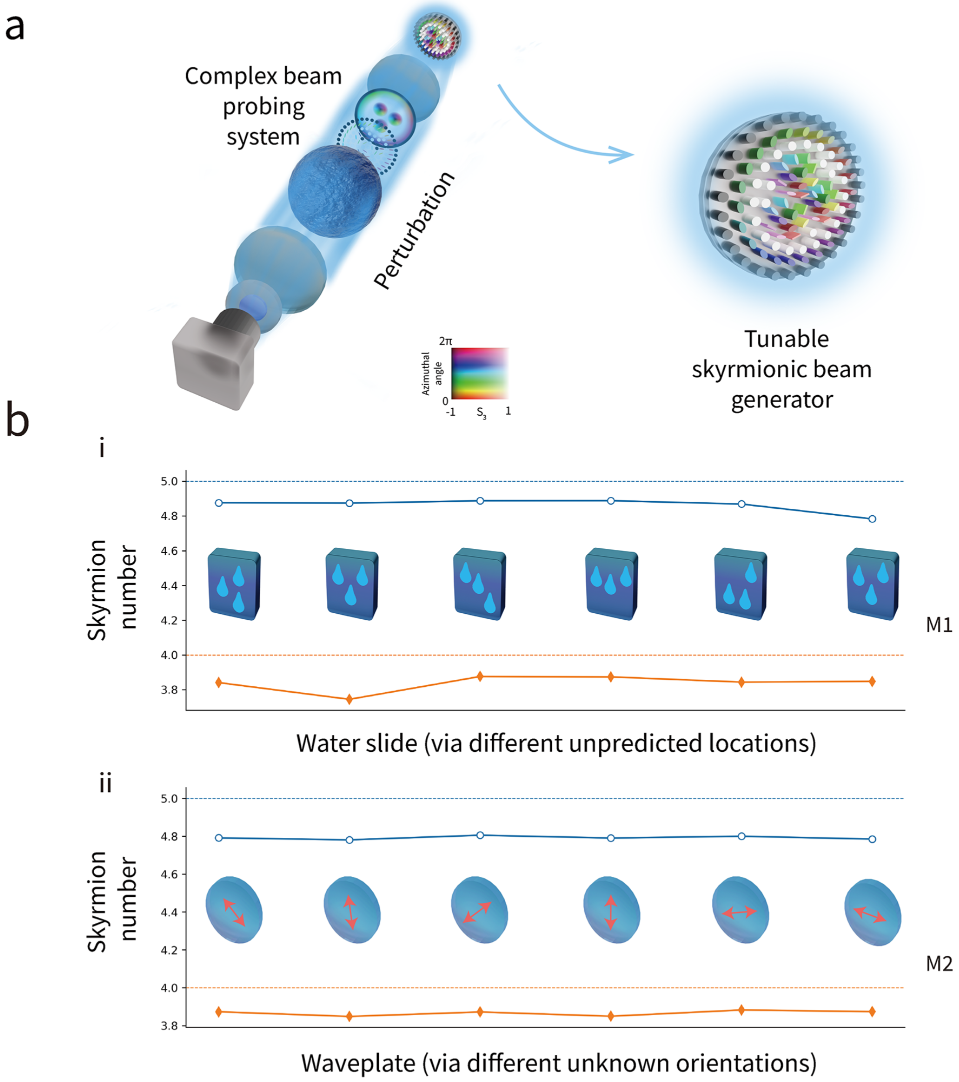

Supplementary Figure 2: Generation and topological protection of high-order skyrmionic beam and skyrmion bag. (**a**) A complex beam probing system under various perturbations. The output beams were recorded by a Stokes polarimeter to characterise the spatially varying SoP and a schematic of the tuneable skyrmionic beam generator. (**b**) Topological protection of high-order skyrmion and skyrmion bag was tested under isotropic (M1) and anisotropic (M2) perturbations. M1: a few-micron thick water layer between two glass slides; M2: an unknown waveplate. (i) shows skyrmion number variations across six positions of M1, and (ii) presents results from M2 with varying waveplate orientations. The calculated skyrmion numbers (y-axis) and field profiles for each position (x-axis) are depicted.

Supplementary Note 3

The tunability of the analysing channel is important, as different applications require different optimisation strategies for SoP sensing. For instance, in certain pathological imaging scenarios, the measurement precision of circular SoP is more important than its linear counterpart [18], whereas linear SoP is given more consideration in certain material characterisation applications [19]. Here the optimised FPUs designed for PO configuration (circularly SoP optimised) and CO configuration (linearly SoP optimised) are illustrated (see Supplementary Figure 3a). Detailed comparison between PO and CO is provided in Supplementary Method 4.

To address various sensing requirements, a polarisation scanning system (Supplementary Figure 3a) utilizing the tuneable PSA adapts between PO and CO configurations. The Mueller matrices of the matter for both configurations are shown in Supplementary Figure 3b, with experimental results aligning well with theoretical results. Decomposed retardance images of both biomedical and archaeological samples are also presented. The full SoP retrieval capability of each FPU is demonstrated in Supplementary Figure 3c using physical inference techniques (see Supplementary Method 4). Additional statistical analyses are presented in Supplementary Figure 3d, which shows the distribution and variance of the measured parameters, and Table S1, which compares the Stokes parameters (S_1, S_2, S_3) obtained using our proposed methods against those measured by a standard Stokes polarimeter (serving as ground truth, GT). To ensure a fair statistical comparison, we use 15 ROIs as an example. The results show approximately $\sim$1% variation between the proposed method (ROI 15) and the GT for both biomedical and archaeological samples at two different zones (Zone 1 and Zone 2), confirming its high precision and reliability. A more comprehensive and detailed validation analysis comparing the CO and PO methods, together with their respective GTs across various sample types, will be the focus of our future work.

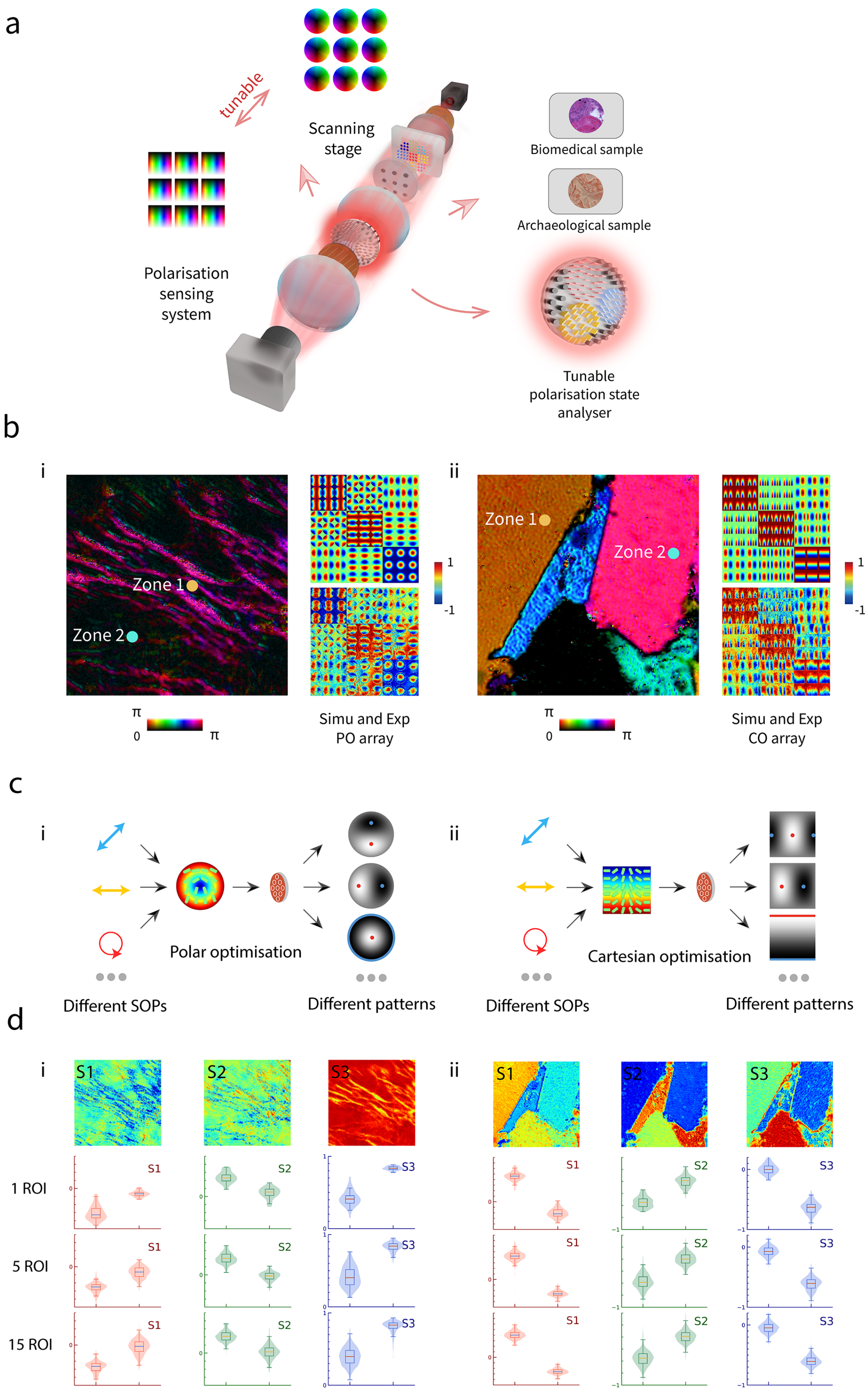

Supplementary Figure 3: Adaptable optimised FPU imaging with the new tuneable optical analyser for biomedical and archaeological sensing. (**a**) A scanning polarimetric imaging system with the PSA, adaptable between PO and CO configurations to suit varying biomedical and archaeological sample sensing requirements. (**b**) The retardance images from PO-optimised analysis of fibrotic tissue (i) and CO-optimised analysis of crystalline structures in an archaeological sample (ii), with simulated and experimental Mueller matrices for

both arrays. In the HSL color map used, the hue (H) represents the fast axis orientation; the lightness (L) reflects the retardance of the measured area; and the saturation (S) is the constant 1. (**c**) PO (i) and CO (ii) units with different input SOPs (linear horizontally polarised, linear 45° polarised and circular polarised light), and different intensity distributions obtained after the fixed circular polariser. The blue and red dots/lines represent the location of the darkest and brightest points, respectively. (**d**) Stokes vector images for the biomedical sample under PO (i) and the archaeological sample under CO (ii). Statistical analysis of S1, S2, and S3 components is shown across multiple regions of interest (ROIs) within designated zones from (b).

Supplementary Table 1: Comparison of Stokes parameters from the proposed method and GT.

	**Biomedical sample**					
	Zone 1			*Zone 2*		
	S1	S2	S3	S1	S2	S3
**ROI 15**	-0.2631	0.2032	0.4078	-0.0201	0.0231	0.8372
**GT**	-0.2648	0.2092	0.3970	-0.0275	0.0173	0.8257
	**Archaeological sample**					
	Zone 1			*Zone 2*		
	S1	S2	S3	S1	S2	S3
**ROI 15**	0.4069	-0.7652	-0.0457	-0.2637	-0.5978	-0.6011
**GT**	0.3945	-0.7502	-0.0492	-0.2509	-0.6069	-0.5903

Supplementary Note 4

Here we demonstrate the sensor-based O-AO method using the compound modulator (Supplementary Figure 4a), offering flexibility to adapt to different correction methods based on system requirements. Detailed correction procedures are provided in Supplementary Method 5.

The configuration of the focusing system for proof-of-concept demonstration is shown in Supplementary Figure 4a, where the O-AO corrector enables validation of sensor-based method through a Mueller matrix polarimeter and sensorless method via focal spot images. The detailed sensor-based correction procedure is described in Supplementary Figure 4b and Supplementary Method 5, providing a clear step-by-step explanation of the process, ensuring accurate and consistent aberration correction throughout the system.

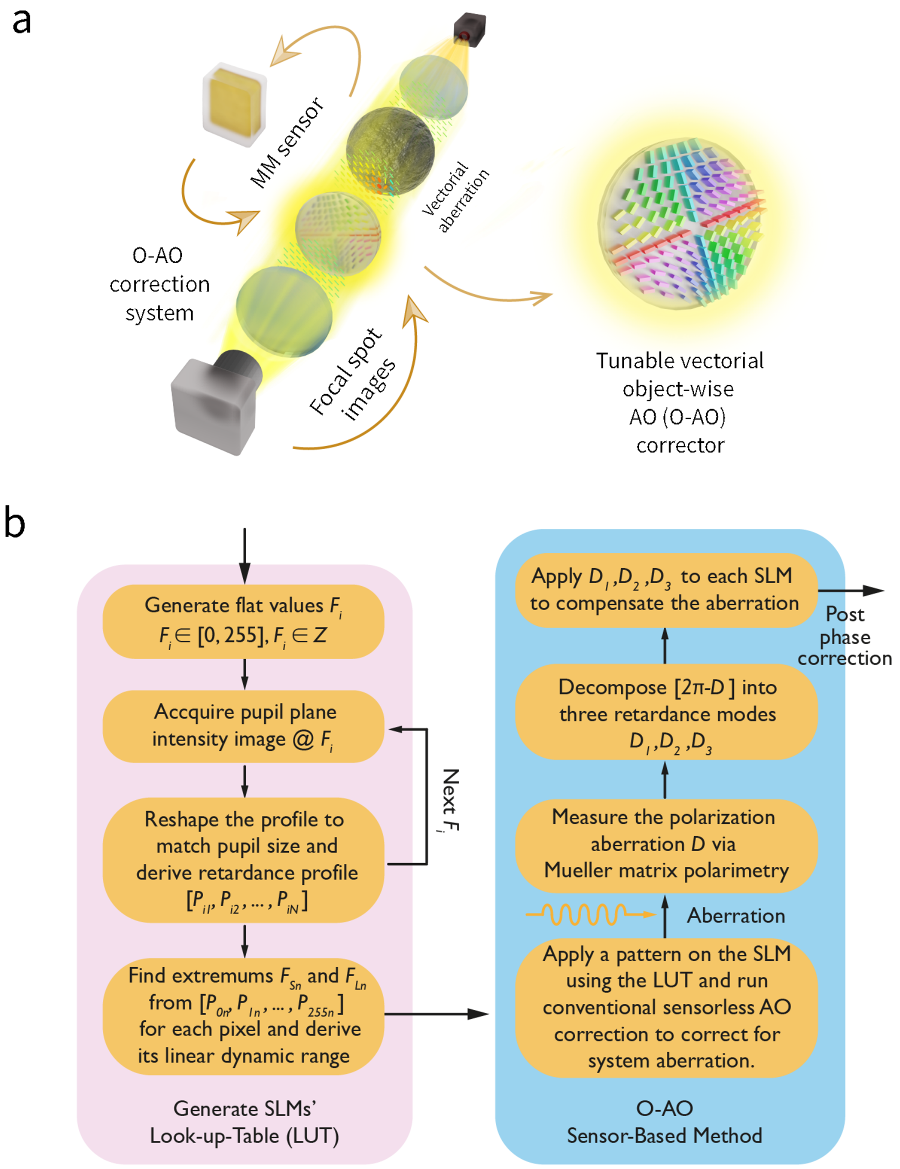

Supplementary Figure 4: Sensor-based O-AO correction using the new beam corrector. (**a**) An optical focusing system with induced aberrations, utilizing the O-AO corrector to validate sensor-based (through a Mueller matrix polarimeter) and sensor-less (through focal spot images) O-AO methods. (**b**) Flowchart summarising the steps for sensor-based O-AO method. The left side outlines the initial SLM calibration process for LUT generation, while the right side details the steps for implementing the sensor-based O-AO correction.

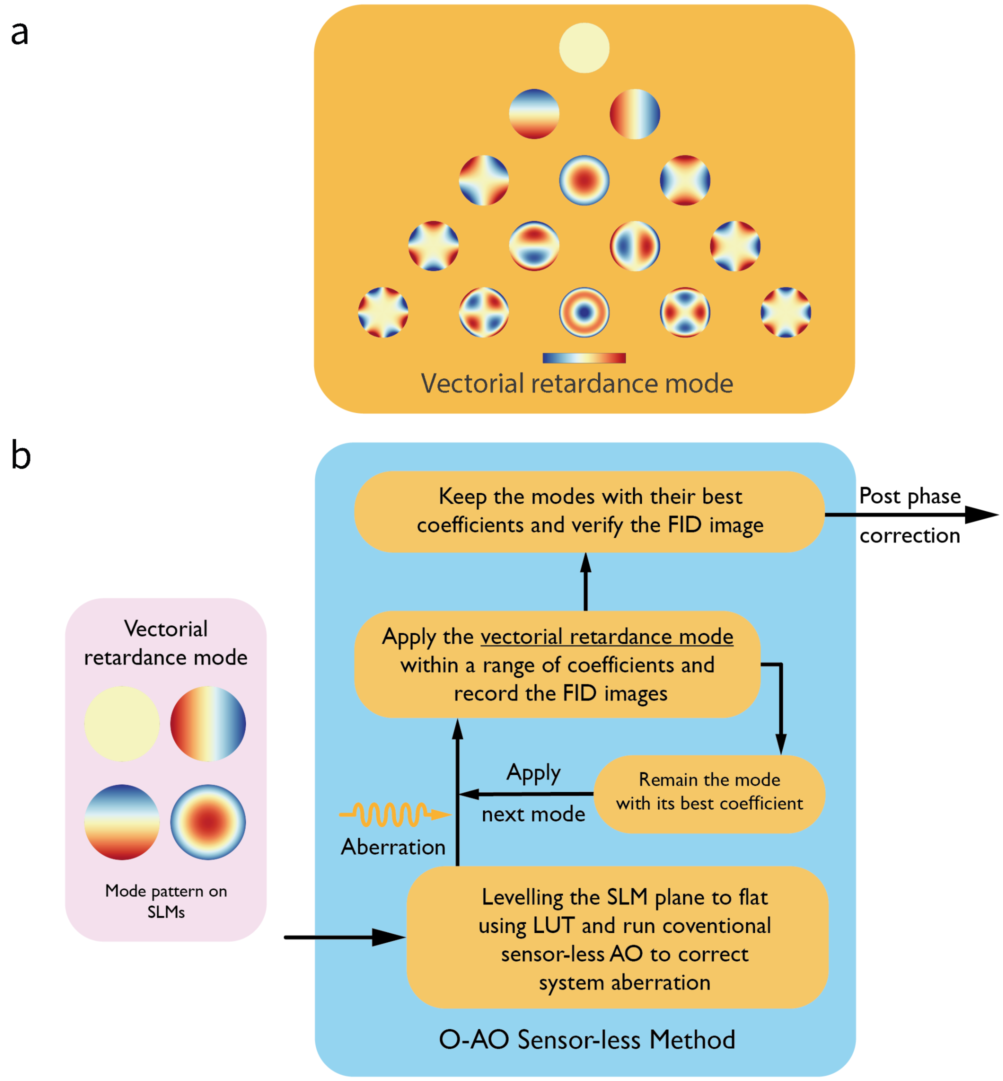

Supplementary Figure 5: Vectorial retardance modes and flow chart summarising the steps for sensorless O-AO method. (**a**) The first 15 vectorial retardance Zernike modes are designed within a range of $[-\pi, \pi]$. These patterns are arranged following Noll's indexing convention. (**b**) The main sensorless O-AO loop utilising the designed vectorial retardance modes is demonstrated.

Supplementary Note 5

Additional examples of how low-functionality devices can be combined to synthesize various types of structured matter, including spatially varying arbitrary retarders, diattenuators, and depolarisers, are shown below.

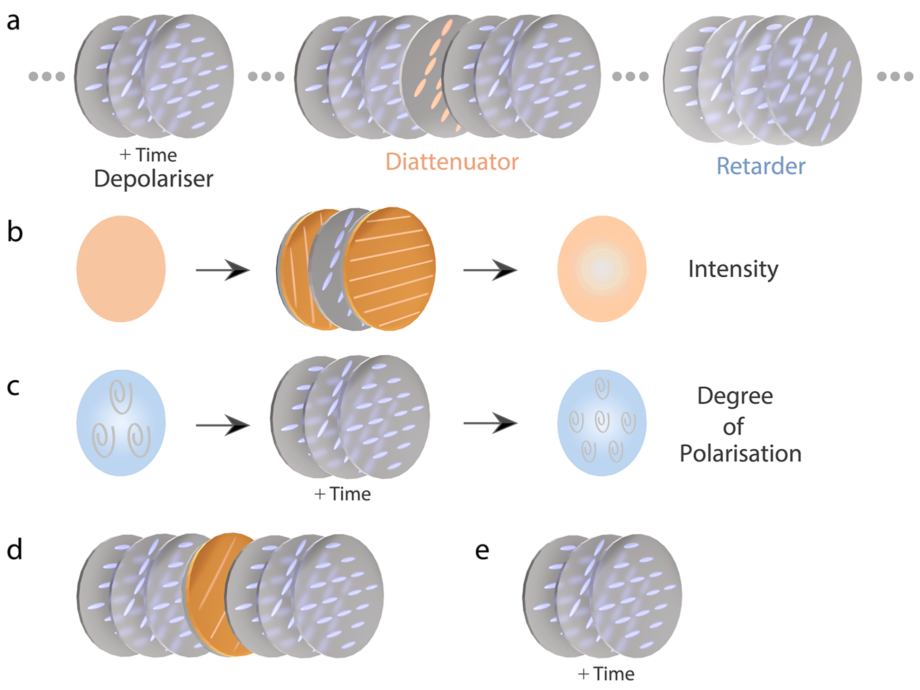

Supplementary Figure 6: Extended cascades and their functionalities. Some implementation possibilities of the cascades that were mentioned in the Discussion are given here. (**a**) General model. From right to left, the first four form an arbitrary retarder array, the middle seven form an arbitrary diattenuator array (the one in yellow represents an SLM filled by liquid crystals blended with an anisotropic dye), and the latter three form a depolariser array in conjunction with time manipulation. (**b**) Pixelated intensity manipulation via a vertical linear polariser, a modulator, and a horizontal linear polariser. (**c**) Pixelated depolarisation manipulation via a cascade of three modulators. (**d**) Cascaded modulators that can form an arbitrary diattenuator (infinite extinction ratio). The first and last three modulators are used to form arbitrary retarders, and the linear polariser in between is used to provide diattenuation. (**e**) Cascaded modulator to form the basis for a depolariser. More information and manipulation mechanisms can be found in Methods 6, 7, 8.

Supplementary References

[1] S. Gao, *et al.*, *Physical Review A* **102**, 053513 (2020).

[2] Y. Shen, *et al.*, *Nature Photonics* **18**, 15 (2022).

[3] D. H. Goldstein, *Polarized light* (CRC Press, 2010).

[4] N. Manton, P. Sutcliffe, *Topological Solitons* (Cambridge University Press, 2004).

[5] N. Nagaosa, Y. Tokura, *Nature Nanotechnology* **8**, 899 (2013).

[6] C. He, *et al.*, *Optica* **9**, 1109 (2022).

[7] C. He, J. Antonello, M. J. Booth, *eLight* **3**, 23 (2021).

[8] M. J. Booth, *Light: Science & Applications* **3**, e165 (2014).

[9] K. M. Hampson, J. Antonello, R. Lane, M. Booth, *Sensorless Adaptive Optics* (2020).

[10] D. Debarre, M. J. Booth, T. Wilson, *Optics Express* **15**, 8176 (2007).

[11] von F. Zernike, *Physica* **1**, 689 (1934).

[12] R. J. Noll, *Journal of the Optical Society of America* **66**, 207 (1976).

[13] J. Antonello, *et al.*, *Journal of the Optical Society of America A* **29**, 2428 (2012).

[14] A. Peinado, A. Lizana, J. Campos, *Optics Letters* **39**, 659 (2014).

[15] D. Marco, *et al.*, *Scientific Reports* **11** (2021).

[16] S.-Y. Lu, R. A. Chipman, *Journal of the Optical Society of America A* **13**, 1106 (1996).

[17] Y. Ma, *et al.*, *Journal of Optics* **26**, 065402 (2024)

[18] J. Chang, *et al.*, *Journal of Biomedical Optics* **21**, 56002 (2016)

[19] M. Cui, *et al.*, *Archaeometry* , 1 (2024)

[20] Y. Dai, *et al.*, *Optical Express* **27**, 35797 (2019)

[21] Q. Hu, *et al.*, *Light Sci Appl* **12**, 270 (2023)
